# Chemical Sensor Platform for Non-Invasive Monitoring of Activity and Dehydration

**DOI:** 10.3390/s150101479

**Published:** 2015-01-14

**Authors:** Dmitry Solovei, Jaromír Žák, Petra Majzlíková, Jiří Sedláček, Jaromír Hubálek

**Affiliations:** 1 Centre of Sensors, Information and Communication Systems, Faculty of Electrical Engineering and Communication, Brno University of Technology, Technická 3058/10, CZ-61600 Brno, Czech Republic; E-Mails: soloveyd@tut.by (D.S.); xzakja07@stud.feec.vutbr.cz (J.Z.); businova@feec.vutbr.cz (P.M.); xsedla44@stud.feec.vutbr.cz (J.S.); 2 Central European Institute of Technology, Brno University of Technology, Technická 3058/10, CZ-61600 Brno, Czech Republic

**Keywords:** sensor, dehydration monitoring, potassium ion selective electrode, data acquisition, humidity, urine, sweat sensor, perspiration monitor

## Abstract

A non-invasive solution for monitoring of the activity and dehydration of organisms is proposed in the work. For this purpose, a wireless standalone chemical sensor platform using two separate measurement techniques has been developed. The first approach for activity monitoring is based on humidity measurement. Our solution uses new humidity sensor based on a nanostructured TiO_2_ surface for sweat rate monitoring. The second technique is based on monitoring of potassium concentration in urine. High level of potassium concentration denotes clear occurrence of dehydration. Furthermore, a Wireless Body Area Network (WBAN) was developed for this sensor platform to manage data transfer among devices and the internet. The WBAN coordinator controls the sensor devices and collects and stores the measured data. The collected data is particular to individuals and can be shared with physicians, emergency systems or athletes' coaches. Long-time monitoring of activity and potassium concentration in urine can help maintain the appropriate water intake of elderly people or athletes and to send warning signals in the case of near dehydration. The created sensor system was calibrated and tested in laboratory and real conditions as well. The measurement results are discussed.

## 1. Introduction

Monitoring of important biological processes of the human body is one of the current concerns of personal medicine to ensure healthy and long life expectancies. From this point of view, interest in wearable portable and hand-held sensors to provide health monitoring functions has increased in the last decades. These sensors are currently in the form of developmental point of care platforms. A number of such sensors with a controlling device can be integrated as a wearable Wireless Body Area Network (WBAN), which can be used for computer-assisted monitoring of chronic diseases, diabetes, asthma, heart attacks or dehydration state as well [1–8].

Water is the most important substance for the human organism and the quantity of fluids influences the correct function of all body cells [6]. The water metabolism of body is controlled through water intake and water output. The most significant output is urination, therefore it has to be well monitored and used for calculation of the amount of water loss. During humans' daily movement, sport activities and fitness, a large amount of fluid excretes through the skin in the form of sweat and by breathing. The human organism can produce about 600 mL of sweat per day in ordinary conditions during standard life activity. Approximately the same amount of fluids is lost through breathing. Besides, the monitoring of excreted fluid is very important for elderly people, sick persons and people living in areas with high temperature. In cases of intense stress, 0.33 L/h of sweat can be produced at an ambient temperature of 27 °C while 0.58 L/h is produced at 35 °C [9].

Dehydration of the organism leads to disruption of the cells and brain work and reduces the functioning of metabolic processes. The first symptoms of dehydration appear at the loss of about 2% of the body fluids [6,10]. Existing methods for measuring dehydration states are intrusive or invasive which makes their use in everyday life complicated. One of the possibilities for non-invasive dehydration monitoring is determination of potassium concentration in urine [11]. This concentration is reciprocally proportional to total amount of water in the human body. Standard values vary between 100 mg/L and 2000 mg/L in most cases [12]. The concentration of potassium in urine can be measured by a standard ion-selective electrode and processed in any type of medical device or data logger and recalculated as an organism hydration value [13]. Determination of this value is not accurate due to depending ordinary potassium concentration on gender, age or condition of the monitored person [14]. Long term measurements have to be performed separately for basic potassium concentration value determinations of each monitored person.

Sweating process monitoring can be interpreted as a personal activity and it can be determined through the measurements of humidity level close to skin surface as perspiration rate. Activity is also related to water metabolism and it can impact dehydration significantly, but it is difficult to determine dehydration from these data. Therefore, an activity monitor is an additional sensor helping to better understand data from urine sensor and giving feedback to a person in time to avoid dehydration. The sensor should be safe for human body and stable in a salt atmosphere during a long period of time (hours, days, months). Metal oxide semiconductor sensors, which also have fast response time, low drift of the parameters and don't have hysteresis, meet these requirements [15–22]. Recently, with the development of nanotechnology, arrays of nanostructured oxide (nanodots, nanopillars, nanowires, nanopores) systems of valve metals attract increasing popularity as a basis for a sensitive film of different metal-oxide sensors [15]. The main valve metals usually used in preparation of nanostructured oxides for sensor applications are titanium, tungsten, zirconium, niobium, tin, *etc.* [16–22]. Nanostructures prepared on the surface with very simple technique present promising properties for sensing ions in vapors and gases and also for electronic components. The advancing development of science in the area of nanoelectronics can significantly reduce the size of such sensors and their power consumption, which opens the possibility of using them as *in-situ* or wearable sensors, e.g., for human biological processes monitoring [23–25] close to the skin or vapors, humidity and gases measuring in mobile applications. One of the more promising materials for humidity sensor applications is micro- and nanostructured titanium oxide film, which can be fabricated in the form of nanowires [26,27], nanofibers [28,29], nanocrystalline thin films [30], nanocomposite thin films [31–33], microstructures TiO_2_ films [34], titanium oxide nanotubes [35], nanopowders [36,37], nanocolumns [38] and nanostructured films produced by glancing angle deposition [39] or sol-gel [30] techniques.

In this work, we propose and develop a sensor platform for non-invasive dehydration detection and monitoring. It is extremely important for elderly people or athletes to prevent serious health problems or sudden death [40]. The platform uses a combination of water intake with excreted fluid monitoring and determination of potassium concentration in urine for precise assessment. This combination is used due to practical reasons because standalone non-invasive measurements are not accurate enough. The individual sensor devices are connected to the developed Wireless Body Area Network (WBAN) for online collection of measured values from both devices. The fluids excreted through the skin during active or passive day activity are measured by two humidity and temperature sensors located near the skin and in ambient air. Water outtake is calculated from a known distance and measured humidity gradient. For near skin measurement, a new TiO_2_ sensor was developed. This sensor has increased sensitivity in the high humidity conditions which occur near the human skin. It uses sensitive element based on array of titanium oxide nanopillars formed by an electrochemical anodizing method.

The concept of wireless sensor platform which combines mentioned sensors for this purpose, as well newly developed TiO_2_ sensor for relative humidity measurement, is innovative. Therefore, the major part of this article is primarily focused on TiO_2_ sensor development and fabrication due to its novelty and benefits.

## 2. Experimental

### 2.1. Development of the Wireless Dehydration Monitoring System

**Figure 1.**
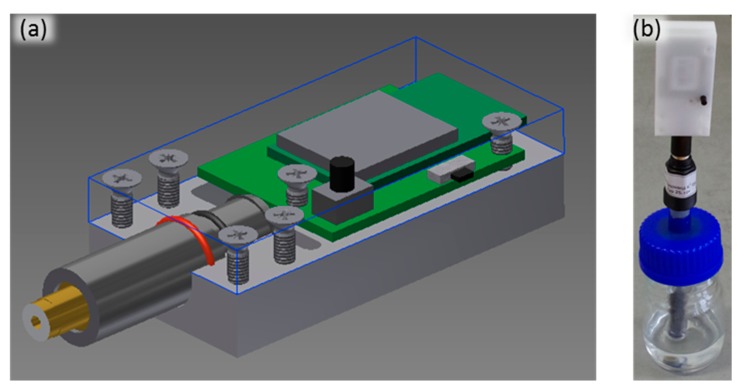
(**a**) Design of the developed wireless device and (**b**) the whole system set-up for automated measurement of potassium concentration in urine.

The dehydration monitoring system is based on an electrode sensitive to potassium and a device for measurement and wireless data transfer. Potassium concentration is measured by a standard potassium ion selective electrode obtained from Elektrochemické detektory s.r.o. (Trutnov, Czech Republic) with potentiometric output according to the calibration curve. The voltage is converted into data values in the form of digital numbers by a standalone wireless battery powered electronic circuit after each measurement (see Figure 1a). The measurement vessel, electrode and electronic device form a compact system set-up as can be seen in Figure 1b. Measured data are transferred using a WBAN and collected in an internet database where they can be read, viewed or analyzed by PC or smart phone. Additional values like personal information can be inserted manually by the user.

#### Measurement of Potassium Concentration in Calibration Solutions and Real Urine Samples

The potassium ion selective electrode had been conditioned before use in a 0.5 mmol/L KCl (PENTA, Prague, Czech Republic) aqueous solution for 1 h. The calibration of the system was performed using KCl solutions with a concentration in the range between 1 mg/L and 10 g/L with addition of urea in the concentrations of 0%, 0.5%, 1%, 1.5% and 2% (typical values of urea concentration in urine [12]). All solutions were prepared using Milli-Q water (Millipore, Billerica, MA, USA). Real samples of urine were collected from ten persons to verify the functionality of the developed system. Determination of potassium was performed directly in the urine samples without any dilution.

### 2.2. Development of the Wireless Activity Monitor

The activity monitor is based on determination of skin perspiration rates during daily activities. Sweating depends on the effort expended in activities. Humidity close to the skin increases with the effort of the person and ambient conditions. It is necessary to have two humidity and two temperature sensors with a certain distance between them and the skin in measurements to calculate perspiration rate.

The water transport through the skin, the so called transepidermal water loss (TEWL, g/m^2^/h), calculated with a well-known mathematical model [41,42] is used in the activity monitor. This model is based on the estimated vapor pressure gradient of the difference between RH on the surface of the skin and at the certain distance, given by Equation (1):

(1) $$\frac{1}{S}\cdot \frac{dm}{dt}=-D^{\prime }\cdot \frac{\partial p}{\partial x}$$

where 1/*S*·(d*m*/d*t*) is the evaporation rate (g/m^2^/h), *D′* is the constant 0.67 × 10^−3^ (g/m/h·Pa), (∂*p*/∂*x*) is the vapor pressure gradient in (Pa/m). From this equation, we can calculate the rate of sweat evaporation close to the skin surface and then interpolate the total amount of evaporated water as the result of integration over time.

#### Fabrication of the Nanostructured TiO_2_ Chemo-Resistive Humidity Sensor

The nanostructured TiO_2_ sensing layer was synthesized by using a local electrochemical anodization process through a porous alumina template. A Ti/Al bilayer was deposited by magnetron sputtering onto an oxidized silicon substrate. The titanium layer was 200 nm thick and the layer of aluminum had a thickness of 1 μm. Each sample with the size of about 7 mm × 7 mm was placed below a specially designed electrochemical cell with a 16 mm^2^ anodization window. A Haake DC-30 Heating Circulator (Thermo Fisher Scientific, Waltham, MA, USA) was used for temperature stabilization of the anodization process. Millipore water was used for the preparation of electrolytes and sample washing. Anodization of aluminum and titanium layers was performed in a 0.9 M aqueous solution of oxalic acid in the galvanic static mode with the current density of 4 mA/cm^2^ and the voltage of 30 V on stationary stage of porous alumina growing. When the anodization front reached the titanium underlayer and all aluminum was fully consumed, the anodization voltage began to increase and its growth continued up to a certain limited value at 37 V. Then the anodizing mode was automatically switched to the constant voltage mode for electrochemical anodization of titanium underlayer to continue. The titanium anodization process took about 40 min. After reaching the constant minimum value of anode current (less than 50 μA/cm^2^) [43] the process was switched off and the sample was washed and dried. A Keithley 2400 programmable power supply (Keithley Instruments, Inc., Cleveland, OH, USA) controlled by LabVIEW (National Instruments, Austin, TX, USA) was used as the anodizing system.

**Figure 2.**
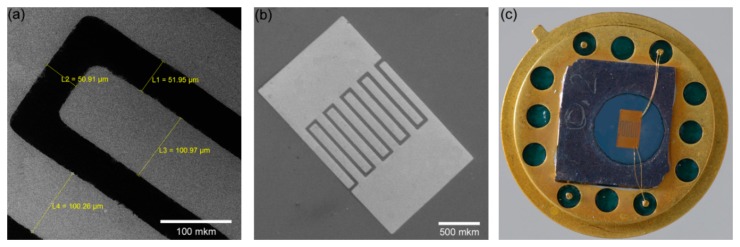
Detail of the sensing part microstructure: (**a**) electrode gap; (**b**) interdigitated electrodes and (**c**) sensing chip packaged on TO-8.

After completing the anodizing process, the porous alumina matrix was selectively removed in 20 g/L CrO_3_, 35 mL/L H_3_PO_4_ solution kept at 65 °C for 15 min thus opening the array of titanium oxide nanopillars. A photoresist mask for the formation of gold electrodes that provide electrical contact to the sensor structure was prepared by photolithographic techniques on the surface of Ti/TiO_2_ layer. The mask was made in the form of an inter-digital structure with an electrode width of 100 microns and spacing of 50 microns (Figure 2a). Electrochemical deposition of gold, which was conducted in an aqueous solution of K[Au(CN)_2_] with the addition of H_3_BO_3_ was performed into the prepared mask at 50 °C in a constant current mode with the density of about 2 mA/cm^2^ for 5 min and with the voltage drop on an electrochemical system at 1.3–1.1 V. The thickness of the gold layer was about 300 nm. After performing the electrochemical deposition of gold, photoresist etching was carried out in acetone for 10 min. Then, the samples were annealed in an air atmosphere at 600 °C for 2 h (Figure 2b). The sensor fabrication process was ended by dicing into chips and packaging the microchips, assembly on a standard TO-8 metal package and wiring using ultrasonic wire bonding (Figure 2c).

### 2.3. Development of the Wireless Perspiration Sensor System

**Figure 3.**
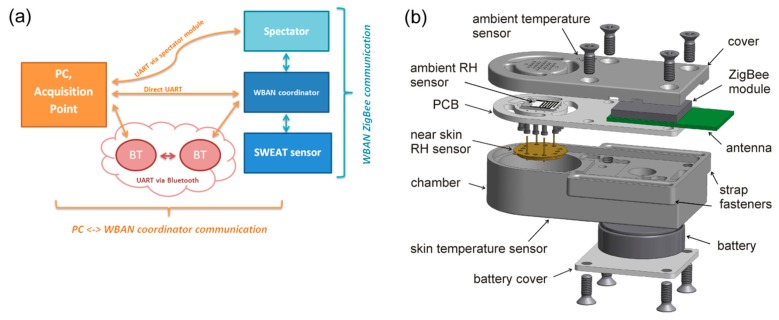
Sensor system: (**a**) wireless data transmission schematic and (**b**) sensor 3D design model.

**Figure 4.**
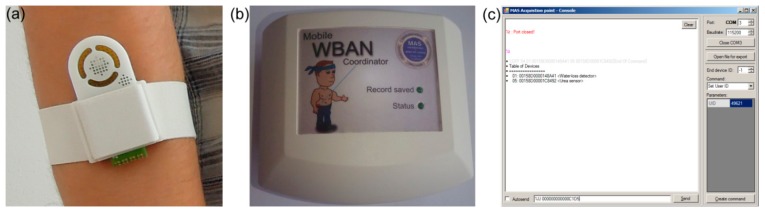
WBAN system: (**a**) wireless perspiration sensor; (**b**) WBAN coordinator and (**c**) software for monitoring of data transfer to acquisition point.

An on-body wireless perspiration sensor system (see Figure 3a) for body fluid output monitoring through the skin was developed. The device is composed of two resistive relative humidity sensors, two temperature sensors, a wireless electronic module for measurement, calculation and data transfer, and a battery power supply (see Figure 3b). The humidity sensor based on a nanostructured TiO_2_ layer was used for measuring of relative humidity (RH) close to the skin surface at a distance of 5–7 mm (skin RH sensor). The second commercial resistive humidity sensor HS-15P is located on the opposite side of the sensing unit. This sensor was intended to measure ambient relative humidity (ambient RH sensor). The DS18B20U sensor with digital output has been used for skin temperature measurements. All components of the sensing unit are integrated in the box, which can be fixed on the arm of the person (see Figure 4a). Changes of resistance of both humidity sensors are converted into voltage values by a OPA2030 low power rail-to-rail operational amplifier which works as a high-resistance to voltage converter. The generated voltage is processed directly to the JN5148 ZigBee module which is equipped by 12 bit SAR analog to digital converters. This wearable device is permanently connected to the wearable WBAN coordinator (see Figure 4b) and PC with special application (see Figure 4c).

### 2.4. Films Characterization, Humidity Measurements and Perspiration System Test Methodology

The surface and cross section morphology of the nanostructured TiO_2_ layers were observed by scanning electron microscope Mira II LMU (Tescan, Brno, Czech Republic) with an operation voltage between 15 and 25 keV. X-ray diffraction (XRD) analysis was performed at the Department of Condensed Matter Physics (Masaryk University, Brno, Czech Republic) using a laboratory assembled diffractometer equipped with Cu X-ray tube, Siemens high voltage generator, Soller slit, graphite monochromator, and a Radicon scintillation detector. Before analysis, all samples were washed in flowing Millipore water for a few minutes three times and then dried in a stream of warm air.

Relative humidity (RH) measurements of the developed nanostructured sensor were done by using saturated salts solutions providing stable air humidity near the surface. These solutions include: LiCl (relative humidity (RH) of 11%), MgCl_2_ (RH of 33%), K_2_CO_3_ (RH of 43%), Mg(NO_3_)_2_ (RH of 57%), NaCl (RH of 75%), KCl (RH of 85%), and K_2_SO_4_ (RH of 97%) [44]. The sensor output voltage was recorded after 3 min of value stabilization. Before RH measurements nanostructured TiO_2_ sensors were aged in the atmosphere of 97% RH vapor for 8 h.

The system test was started by calibration of both humidity sensors (the developed TiO_2_ and commercial HS-15P) by using saturated salt solutions. After the calibration, necessary adjustment of electronics was done because of different impedance values of the new and commercial sensors. Then, the portable part of the system was tested with the help of four volunteers during standard life activity and sport exercises. During testing each volunteer wore the perspiration sensor module on the arm (see Figure 4a) and the coordinator unit on the belt. Test time was varied between 4 and 10 h. After testing, the data from coordinator unit were transmitted to the acquisition point (AP) on the personal computer and testing diagrams with necessary calculations and analysis were done. Power consumption of the sensor devices was evaluated for battery life estimation during testing as well.

## 3. Results and Discussion

### 3.1. Development and Testing of the Wireless Dehydration Monitor

**Figure 5.**
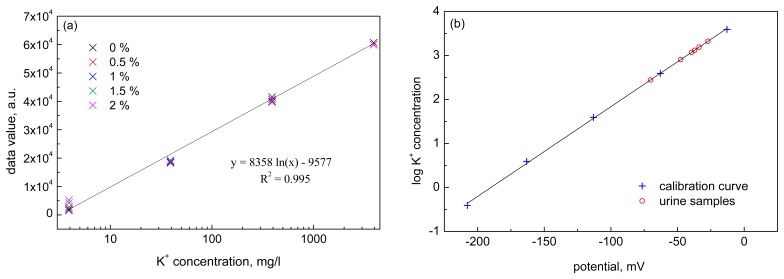
(**a**) Calibration of the sensor system in the KCl solutions with the concentration in the range 4–4000 mg/L with addition of urea in the concentrations of 0%, 0.5%, 1%, 1.5% and 2% and (**b**) potassium concentration determined in real urine samples fitted to calibration data.

Automated measurement of potassium concentrations in urine by the developed system for real-time data acquisition was used for dehydration detection/monitoring. The whole system was created, calibrated and tested under the laboratory and real conditions. Figure 5a shows the average calibration curve obtained for potassium solutions with different urea concentrations. As can be seen, good linearity of the calibration curve was achieved in the studied potassium concentration range with correlation coefficient of 0.995. Furthermore, the interference of urea was found to be practically negligible in the studied range of urea (0.5%–2%). Figure 5b shows values of potassium concentration determined in real urine samples and their fitting to the calibration curve. It can be seen that a good correlation was achieved between the values measured in real samples and the calibration curve.

### 3.2. Characterization of the Nanostructured Titanium Oxide Film

**Figure 6.**
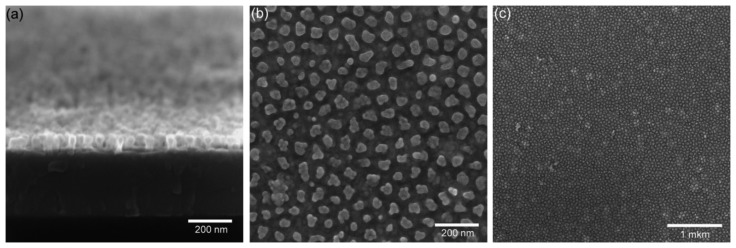
SEM images of sensing part of sensor: (**a**) nanostructured titanium oxide layer; (**b**) top view detail of TiO_2_ nanopillars and (**c**) homogeneous distribution of TiO_2_ nanopillars on large area.

The SEM images (Figure 6) show nanostructured titanium oxide layer obtained by electrochemical anodization process through the porous anodic alumina mask as described above (Section 2.2). As seen from the images, nanostructured titanium oxide film consists of an ordered array of individual pseudospherical pillars separately disposed one to each other, with diameters of the tops between 30 nm and 50 nm. These observed nanopillars are anodic titanium oxide, which had been formed at the original sites of the alumina nanopores, whose mean diameter is 15 nm. Actually, when the titanium oxide nanopillars grow, they penetrate an approximately three times wider region in the alumina barrier layer than the diameters of the alumina nanopores. The number of TiO_2_ pillars corresponds to the number of pores and their packaging density is approximately (2–3) × 10^10^ pil/cm^2^. Each pillar has a smaller lower part diameter and a bigger upper part diameter as seen from cross section image (Figure 6a). The difference between diameters of lower and upper part can be explained by growth of titanium oxide inside of the barrier layer of alumina cells and inside the free space of pores. Inside the alumina barrier layer during transport of Ti and Al ions mixing of titanium oxide with alumina takes place [37] and only small channels (about 10 nm) in the center of alumina barrier layer have more pure TiO_2_ material. After selective removing of the porous alumina layer the mixed part of TiO_2_ is removed simultaneously with the alumina leaving the small diameter lower part of the nanopillars (Figure 6b). In any case, the mechanism of TiO_2_ growth inside of alumina barrier layer is complicated and should be investigated separately. As seen in Figure 6a the height of the nanostructured titanium oxide layer is about 70–75 nm and the spacing step of about 75–80 nm. Such sensing layer morphology, with the height of pillars and distance between pillar centers approximately the same, was chosen for a better sensitivity and fast response of the sensor. In our opinion, in this case water molecules will better penetrate between pillars and absorption-desorption process will work more homogenously on the whole sensing nanostructured surface (Figure 6c).

**Figure 7.**
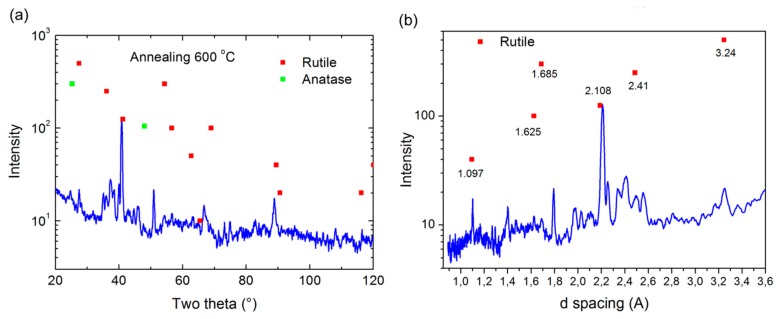
XRD analysis records: (**a**) 600 °C air annealed nanostructured TiO_2_ layer (**b**) d-pacing TiO_2_ crystals in rutile phase.

XRD analysis of phase composition of the thermal annealed TiO_2_ samples was done after anodization and chemical etching of porous alumina mask. Figure 7a shows the XRD pattern of 600 °C air annealed nanostructured TiO_2_ layer, where the nanostructured titanium oxide layer has a few strong peaks of rutile phase located at 2θ angles 41.2° and 89° and few small peaks located at 2θ angles 27.4°, 36.1°, 54.3°, 56.6°, 116.3° and some peak inside of noise as can been seen from diffractogram.

We also detected a small anatase phase located at 2θ angles 25.3° and 48°. We suppose that during thermal annealing process (2 h) all crystals were not transformed from the anatase to the rutile phase configuration. The interatomic d-spacing measurements also revealed that the size of the spacing of the main TiO_2_ crystals in the rutile phase were between 1.097 and 3.240 nm (see Figure 7b). These differences of interatomic d-spacing correspond to the morphology of TiO_2_ pillars. The size of different pillar parts was between 10 nm and 40 nm and as a result rutile crystals were modified from the root to top of the pillar.

### 3.3. Response of TiO_2_ Sensor to Relative Humidity

**Figure 8.**
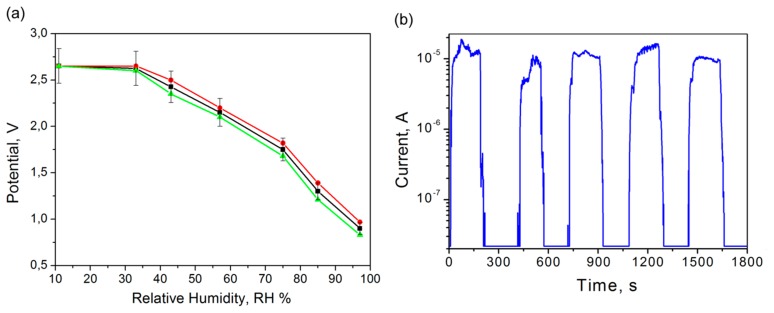
Relative humidity measurements: (**a**) deviation of three sensors responses and (**b**) time response of the sensing layer during changing RH from 11% to 97%.

The relative humidity (RH) measurements of TiO_2_ sensor were performed in a closed chamber filled with saturated salt solutions in the range of humidity from 11% to 97% according to the methodology described above (Section 2.4). A special electronic module was developed for conversion of sensor impedance value into a voltage signal changing from 3 V to 0 V. The maximum of the signal corresponds to the minimal value of RH data and *vice versa* the minimum of the voltage signal matches the maximum RH value. The results of RH measurements for fabricated TiO_2_ sensors are shown in Figure 8.

Responses of three individual TiO_2_ based sensors are presented in the Figure 8a. All responses show identical almost linear curves while the RH is changing. The deviation of data (Figure 8a) is about 7% and the slope of curves is similar. The difference of values between 11% and 33% was not observed due to the very high impedance and the developed impedance-voltage converter, but after 33% it was possible to measure the slowly decreasing voltage. After the measurement of output voltage dependency on RH value, a current change thorough the sensor was measured as a response on the applied fast RH step change between 11% and 97%. The maximal output value range was determined in this measurement. For this purpose, we applied a constant potential of 3 V to the sensor by a power supply and the flowing current was recorded. The results of these measurements are shown in Figure 8b. The level of current for the maximum RH value of 97% is about 10 μA. For the lowest RH value of 11% the current level is about a few tens nA. The maximum time response of the sensor to reach 97% RH from 11% RH is between 40 to 60 s which was verified during long-term testing. The deviation of current data was more than 7%, but in comparison with voltage signals obtained from the first measurement (Figure 8a) the current range cover through three orders of magnitude. In the case of low currents the accuracy is low. Therefore, only voltage measurement was selected for future use in developed devices.

The sensing mechanism of the semiconducting titanium oxide is based on the adsorption of the surrounding water molecules by the nanomorphology surfaces. Adsorption occurs at exposed fivefold coordinated Ti sites in each case with the hydrogen atoms pointing away from the surface. An H_2_O molecule adsorbed in a vacancy would provide a geometrically particularly well-suited adsorption site for O–H interaction and dissociation. The nanostructured morphology of the sensing layer offers more possibilities for the formation of oxygen vacancies which helps improve the adsorption-desorption process as well. Adsorption of water molecules is going on in a few layers and the growth of a multilayer provides the formation of hydronium ions. Hydronium ion interactions between the absorbed species and the bridging oxygen atoms of the TiO_2_ sensing layer are expected to facilitate proton transfer with a corresponding increase of electrical conductivity. This process is reversible at room temperature and depends on the humidity level of ambient air, which means that when the humidity level rises a higher concentration of mobile protons increases the electrical conductivity [33,45,46].

### 3.4. The Wearable Perspiration Monitoring System under Test

**Figure 9.**
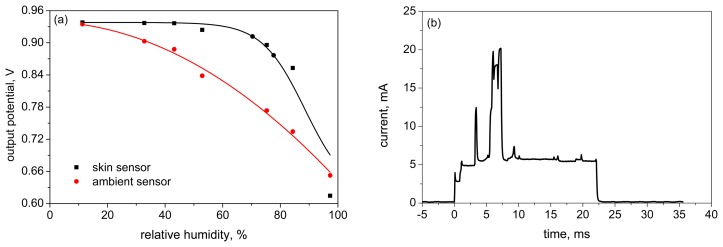
Sensor characteristics: (**a**) the comparison of developed and commercial humidity sensing element response and (**b**) current consumption of the perspiration sensor module during measurement and data transmission.

Before testing the whole wearable system, the calibration of both humidity sensors (nanostructured TiO_2_ based and commercial HS-15P, (Steatite group Ltd., Birmingham, UK)) was done using saturated salt solutions (see Section 2.4). The calibration results presented in Figure 9a show that the signal from the TiO_2_-based sensor has a better sensitivity in the higher humidity region and the commercial HS-15P sensor has lower sensitivity balanced in the whole studied humidity range.

The TiO_2_-based sensor was chosen as skin humidity sensor due to its higher sensitivity in the high humidity environment as confirmed by calibration measurements (see Figure 9a). For ambient air humidity levels, it is not necessary to use the TiO_2_-based sensor due to the lower humidity values, therefore, a commercial sensor HS-15P was used. Also during the adjustment process the level of the voltage signal was decreased in accordance with the analog to digital converter input range. The accuracy of the skin sensor after calibration was determined to be about 10% RH in the range of RH ≤ 50% and 3% RH in the range of RH > 50%. Error drifts over time (about 10% RH) were detected during one month after calibration. In the case of the commercial sensor the maximum error was determined to be about 4% RH in the whole range. Calibration constants were determined for the sensors and these values were actualized in the perspiration sensor device.

The measured current consumption of the perspiration sensor module is shown in Figure 9b (active voltage range from 2.8 V to 3.3 V). The peak on the diagram (20 mA) corresponds to the switching-on of the device and then the current decreased to the value of about 6 mA. Average power consumption was calculated to be at a level of about 620 μW. According to calculations the lifetime of the sensor modules is more than seven months (for CR2477 3 V batteries). The power consumption of the sensor module was decreased by working in the so-called sleeping mode which was interrupted each five seconds (this time could be controlled by the software). During sleep mode, all supply voltages are removed from the analog circuits except the necessary wireless communication.

**Figure 10.**
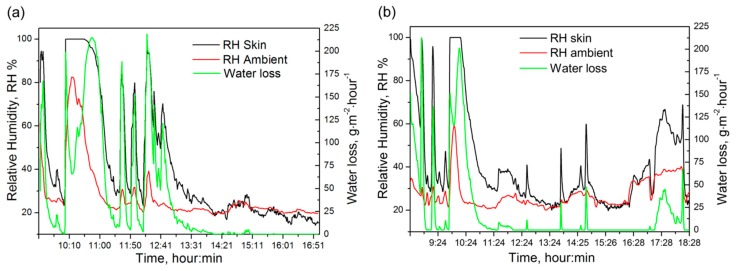
Water loss diagram of volunteers: (**a**) 8-h period of a man and (**b**) 10-h period of a woman.

A typical measurement water loss diagram for a male volunteer during an 8-h period can be seen in Figure 10a and for a female volunteer during a 10-h period in Figure 10b. In the figures, the black line corresponds to data obtained from the TiO_2_ skin sensor, the red line corresponds to data obtained from the commercial ambient sensor and the green line corresponds to the water loss calculation.

Both sensors (skin and ambient) show a high RH value for the male volunteer (Figure 10a) during the first 10 min. As can be seen from the graph, it is the so called set-up period for adaptation of the sensors after storage in dry air. After sensor stabilization the time of low activity of the volunteer is monitored. At 10:00 he jogged for about 40 min and this was a high water loss period. Then during the next few hours the volunteer alternated physical activities and rest periods. Several water loss peaks can be seen in the figure. Finally, after 13:00 the person showed a low activity without significant loss of fluid by sweating. The calculation of water loss for 1.7 m^2^ skin surfaces showed losses of 740 mL of fluids by body perspiration for the 8-h testing time. The skin surface area can be set in the system software according to the age, gender and physical features of the person.

The female volunteer (Figure 10b) had an adaptation time in the first few tens of minutes like in previous case. During the first two hours she underwent a few short training sessions for 10 min and then a long training session for 30 min. On the diagram this corresponds to the two short peaks (black and green lines) around 9 a.m. and one long peak between 10:00–10:30. After the physical activity a short resting period and lunch time (around 12 a.m.) occurred. The second half of the testing time had low physical activity and only short period of time for coffee breaks (a few black line peaks). Finally, the volunteer had physical activity associated with dinner cooking and eating around 6 p.m. The calculation of water loss for 10-h female measurements showed losses of 660 mL of body fluids for the tested period of time. From the comparison of male and female water loss results it is obvious that during the test the man lost more water through sweating than the woman, which depends on the differences of male-female body metabolism for the same level of physical activity.

The analysis of water loss figures showed some influence of the ambient RH sensor from the volunteer sweating during long sport activities. It depends on the distance between the ambient and skin sensors, which is 1.5 cm. However, during long perspiration periods the relative humidity level of the ambient air on the distance of about 2–3 cm around human body can increase. It can be seen from the values of ambient sensor (red line on diagrams in Figure 10). Thus sensor distance adjustment should be done in the future according to the specific environment conditions where the senor will be used.

After testing the system during a rather high and long physical activity, a short time measurement with low physical activity was done. This testing was done for male and female volunteers working for 4-h periods of time sitting in an office with an ambient temperature of about 23 °C. The physical activity of volunteers was very low during the test. The calculations of water loss for this case showed losses of about 40 mL of fluids for a woman and about 65 mL for a man.

## 4. Conclusions

A wearable perspiration system for monitoring of human body water loss by sweating through daily personal activity has been developed and fabricated. The system can be used for fitness activity monitoring indoors or outdoors, and standard daily activity regardless of gender; it has a low power consumption and low weight. The second wireless sensor for dehydration detection/monitoring from potassium concentration in urine with the same parameters was also created. All information obtained during long-term measurements can be stored on any smart mobile device (phone, notebook, tablet PC), stationary computer or hospital server, and sent through any network to medical specialists for monitoring and analysis of water balance (metabolism) of the organism.

A special designed nanostructured titanium oxide sensing layer, fabricated by anodization techniques, was used for measurements of relative humidity level near the human skin surface. The semiconducting TiO_2_ layer consists mostly of a crystalline rutile phase, constructed to ensure fast adsorption—desorption process of water molecules. Based on electrochemical technology, as well as with the use of microelectronic methods, a prototype of the planar relative humidity sensor has been designed and manufactured. The conversion from impedance to the voltage signal of sensor response has linear characteristics with a low response time in the range of relative humidity from 33% to 97%.

The obtained results are promising for further applications in different fields such as fitness, medical or military services and the fabricated system has a low cost and high efficiency.
